# In the Depths of Wash Water: Isolation of Opportunistic Bacteria from Fresh-Cut Processing Plants

**DOI:** 10.3390/pathogens13090768

**Published:** 2024-09-06

**Authors:** Piotr Kanarek, Barbara Breza-Boruta, Tomasz Bogiel

**Affiliations:** 1 Department of Microbiology and Food Technology, Faculty of Agriculture and Biotechnology, Bydgoszcz University of Science and Technology, 6 Bernardyńska Street, 85-029 Bydgoszcz, Poland; breza@pbs.edu.pl; 2 Department of Microbiology, Ludwik Rydygier Collegium Medicum in Bydgoszcz, Nicolaus Copernicus University in Toruń, 9 Skłodowska-Curie Street, 85-094 Bydgoszcz, Poland

**Keywords:** water-borne pathogens, antibiotic susceptibility, fresh-cut processing plants, wash waters, agri-food processing

## Abstract

The fruit and vegetable industry in post-harvest processing plants is characterized by a substantial consumption of water resources. Wash waters may serve as an environment for the periodic or permanent habitation of microorganisms, particularly if biofilm forms on the inner walls of tanks and flushing channels. Despite the implementation of integrated food safety monitoring systems in numerous countries, foodborne pathogens remain a global public health and food safety concern, particularly for minimally processed food products such as vegetables and fruits. This necessitates the importance of studies that will explore wash water quality to safeguard minimally processed food against foodborne pathogen contamination. Therefore, the current study aimed to isolate and identify bacteria contaminating the wash waters of four fresh-cut processing plants (Poland) and to evaluate the phenotypic antibiotic resistance profiles in selected species. Bacteria were isolated using membrane filtration and identified through mass spectrometry, followed by antibiotic susceptibility testing according to EUCAST guidelines. The results revealed that the level of contamination with total aerobic bacteria in the water ranged from 1.30 × 10^6^ cfu/mL to 2.54 × 10^8^ cfu/mL. Among the isolates, opportunistic pathogens including *Enterococcus faecalis*, *Pseudomonas aeruginosa*, *Klebsiella oxytoca*, *Klebsiella pneumoniae*, *Serratia marcescens*, and *Proteus vulgaris* strains were identified. An especially noteworthy result was the identification of cefepime-resistant *K. oxytoca* isolates. These findings highlight the importance of monitoring the microbial microflora in minimally processed foods and the need for appropriate sanitary control procedures to minimize the risk of pathogen contamination, ensuring that products remain safe and of high quality throughout the supply chain.

## 1. Introduction

The fruit and vegetable industry in post-harvest processing plants is characterized by the substantial consumption of water resources. These resources are primarily utilized for pre-treating raw materials during washing and rinsing processes [1]. Utilizing tap water from the distribution network constitutes one of the early stages of vegetable and fruit processing. Its primary objective is to initiate the purification of the raw material from soil particles, pesticides, and undesirable organic matter fragments [2]. Mechanical treatment of fruit and vegetables with the use of turbulent water flow may not be sufficient to reduce microbial contamination levels [3]. Chlorine compounds, due to their widespread availability and ease of application, are the most widely used disinfectants in the reduction of microbial contamination of wash water. However, chlorine is known to react with suspended organic matter, leading to the creation of harmful by-products, including those with carcinogenic properties [4]. Other non-invasive disinfection methods may include ozonation, nanofiltration of water, and the use of peracetic acid [5]. However, currently, some facilities still do not implement disinfection procedures in water used for washing fruits and vegetables. This approach can promote pathogen contamination during production or processing of food products and pose a public health and food safety risk [6,7].

**Figure 1:**
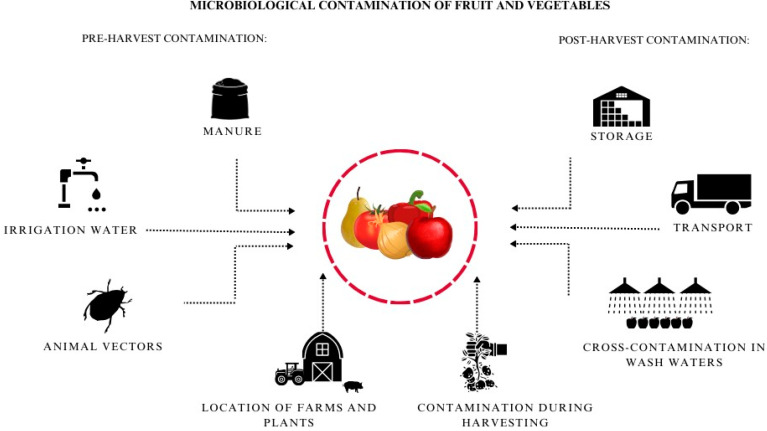
Summary of factors influencing possible pre-release contamination of fruit and vegetables.

There are two types of microbial contamination in vegetables and fruits: pre-harvest contamination and post-harvest contamination (Figure 1). Pre-harvest contamination is most commonly associated with agricultural practices, including fertilization and irrigation [8,9]. One of the significant factors contributing to pathogen contamination is the source of irrigation water (e.g., surface water, groundwater, treated sewage, reservoir water), as confirmed by numerous investigations associated with disease outbreaks caused by foodborne pathogens (such as salmonellosis, listeriosis, and campylobacteriosis) [10,11,12]. Another potential source of contamination is improperly managed irrigation water distribution systems, which create a favorable niche for the development of biofilm, an important source of secondary water contamination [13]. The application of manure, although a common agricultural practice, may also entail risks associated with increased exposure of vegetables to pathogens. Therefore, it is crucial to adhere to proper organic fertilizer application practices, implement a pre-harvest quarantine period, and utilize known sources of supply [14,15]. Another factor that may be a part of pre-harvest contamination is direct zoonotic contamination, resulting from both intensive and extensive livestock production facilities in the vicinity of the crop site. Also, the presence of wild animals and crop pests (e.g., birds, rodents, and insects) can contribute to the transmission of microorganisms to the raw material. The close proximity of different entities is also noteworthy—for instance, the positioning of composting plants, where inadequately managed leachate water can serve as a source of bacterial spread [8,16].

Fruit and vegetables may be affected by microbial exposure in post-harvest contamination already in the harvest stage, e.g., through inappropriate hygiene practices of field workers or inadequate maintenance and preservation of tools and machinery used during the harvesting of the raw material [17,18]. An important aspect requiring careful supervision is the transportation of vegetables and fruits. This includes ensuring the cleanliness of transportation containers and adopting transportation methods that minimize exposure to external factors [19]. Improper pre-processing storage conditions for raw materials (such as lack of warehouse disinfection before storage, excessively high or low temperature, humidity, or ventilation) can induce the development of storage diseases, primarily associated with the activity of filamentous fungi [20]. The final threat contributing to cross-contamination is the washing process of fruits and vegetables without water exchange (or its disinfection between batches). In this case, the washing water can serve as a habitat for microorganisms, either periodically or permanently, especially if biofilm is formed on the inner walls of tanks and rinsing channels.

Currently, there is still a significant risk of foodborne pathogens spreading, particularly for minimally processed food, despite the implementation of integrated food safety monitoring systems in numerous countries [21]. Microbial foodborne diseases result from the direct ingestion of bacteria-contaminated food products, subsequent growth of the microorganisms, and the secretion of toxins that affect physiological host functioning. Another method of exposure is the consumption of food already contaminated with bacterial endotoxins and exotoxins [22,23]. Typical symptoms of foodborne illnesses include abdominal pain, fever, diarrhea, vomiting, nausea, and, in more severe cases, systemic bacteremia and consequent death [24,25].

The development of increasing antibiotic resistance (AMR) and multidrug resistance in bacteria has also been recognized in recent years. Most commonly, these cases are associated with clinical strains posing risks to patients exposed to nosocomial infections. Nowadays, the importance of environmental hot spots in the transmission of antibiotic resistance genes is increasingly emphasized [26]. Enlarged environmental pressure on bacteria, including those with antimicrobial resistance genes, resulting from the supply of antibiotics to the environment, promotes the development (mutational and then vertical) or acquisition (via horizontal gene transfer) of antimicrobial resistance genes. In this case, AMR strains’ development in water or soil may result in secondary exposure to the anthropogenic environment, acting as a specific “feedback loop” [27]. The increased transmission of resistance genes and their spread among pathogens that pose a public health risk is particularly alarming. For example, ESKAPE pathogens (*Enterococcus faecium*, *Staphylococcus aureus*, *Klebsiella pneumoniae*, *Acinetobacter baumannii*, *Pseudomonas aeruginosa*, and *Enterobacter* spp.) may demonstrate high resistance to multiple classes of antibiotics, including both first-line and last-resort options, which can undermine the effectiveness of treatments and impact patient health [28]. Current studies on the antibiotic resistance of strains mainly focus on two fields. The first area involves clinical research, mainly concerned with the resistance of microorganisms in hospital environments. The second research area concerns high-risk points, such as wastewater treatment plants, which are critical links between anthropogenic and natural environments [29,30,31].

There is limited research available on the isolation and phenotypic analysis of antibiotic-resistant microorganisms in washing waters derived from the fruit and vegetable processing sector.

Thus, the objective of this study was to investigate, isolate, and identify bacteria found in the wash waters of four fresh-cut processing plants located in Poland and to evaluate the phenotypic antibiotic resistance profiles in selected species.

## 2. Materials and Methods

### 2.1. Characteristics of Fresh-Cut Processing Plants

**Figure 2:**
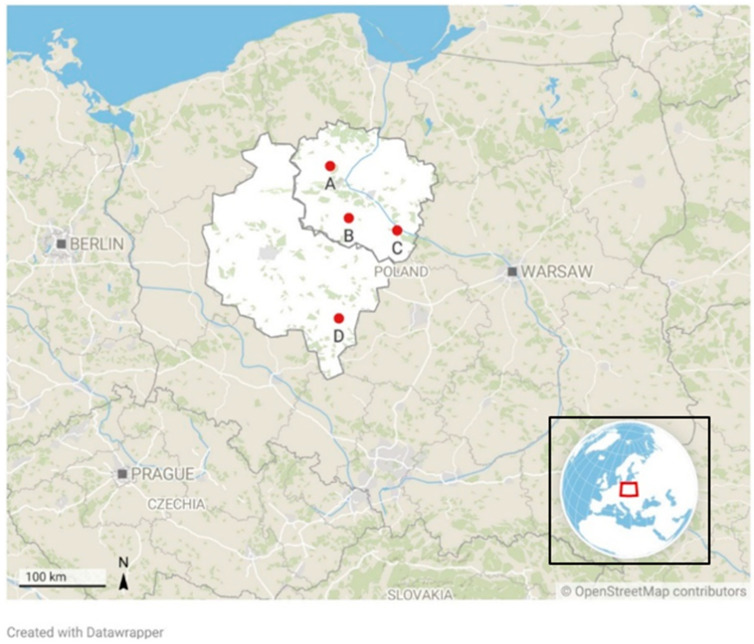
Location of fresh-cut processing plants.

Wash water samples were collected in the full harvesting season, early autumn (September 2022). Post-harvest processing plants are situated in the Kuyavian–Pomeranian (plants: A, B, C) and Greater Poland (plant D) Voivodships (Figure 2) in Poland. These regions are characterized by a highly developed agriculture and food processing industry. The plants process a wide range of fruits and vegetables, both for the domestic market and for export purposes. The plants specialize in ready-to-eat, packaged fruits and vegetables, salads, concentrates, and frozen foods (Table 1). During the interview conducted before sampling, it was noted that no disinfection methods are used during washing at the tested facilities, which may have influenced the contamination of the wash water. All facilities included in this study used tap water, which is regularly tested by sanitary inspection to ensure it meets quality standards.

### 2.2. Wash Water Sampling

Water samples for microbiological analyses were collected under the Polish Standard (PN-EN ISO 19458:2007) [32]. Water samples were taken 3 times, at intervals of 1 h, during the washing of the raw material in the washing tank. Three liters of water were collected from each plant for each analyzed raw material during a single sampling event, using 1 L glass bottles (Chemland, Poland) that had been steam-pre-sterilized. Sampling was conducted under strict sanitary conditions, including the use of facemasks, gloves, disinfection of bottle caps, and storage of samples in insulated transport containers until delivery for analysis. Following collection, the samples were transported to the laboratory of the Department of Microbiology and Food Technology located within the investigated area (Bydgoszcz, Poland). Then, the samples for species identification were pooled to increase environmental representativeness.

### 2.3. Species Identification

After pooling, 3 L of baseline sample was obtained from each washed raw material. Subsequently, microbiological testing of the water was conducted through membrane filtration (filter diameter: 0.22 µm) employing a 3-station filtration unit (Sartorius, Göttingen, Germany). For each bacterial group tested, 100 mL of water was filtered in duplicate. After filtration, the filters were placed on a dedicated microbiological medium. For the total bacterial count test, surface plating was performed by pipetting 1 mL of water onto the medium and spreading it with a spatula. The following groups of bacteria were isolated on dedicated culture media: *Escherichia coli* (and the remaining *Enterobacteriaceae* family) (medium: Agar Endo, Merck; incubation: 24 h at 35 ± 0.5 °C), *Staphylococcus* spp. (Chapman-agar, Merck; incubation: 48 h at 35 °C), *Pseudomonas* spp. (Pseudomonas Selective agar with Pseudomonas CN Selective Supplement, Merck; incubation: 44 ± 4 h at 25 ± 1 °C), *Legionella* spp. (Legionella BCYE-Agar with Legionella Growth Supplement, and Legionella (GVPC) Selective Supplement, Merck; incubation: 10 days at 36 ± 2 °C), *Enterococcus* spp. (Kanamycin esculin azide agar, Merck; incubation: 24 h at 36 °C), and *Salmonella* spp. (SS agar, Merck; 24 h at 36 °C), total aerobic bacterial count (Standard Agar I, Merck). After the incubation period, colonies specific to certain groups of microorganisms were counted to determine colony-forming units (cfu) per 100 mL (and cfu per ml for total aerobic bacterial count). Pure bacterial cultures were then cultured, and species identification was performed by mass spectrometry via MALDI Biotyper apparatus (Bruker Daltonik GmbH, Bremen, Germany) with CE and IVD certification (according to Directive 98/79/EC).

### 2.4. Evaluation of Phenotypic Antibiotic Susceptibility of Selected Strains

Antimicrobial susceptibility testing of the selected species was performed and interpreted by the standard disc-diffusion method, according to the European Committee on Antimicrobial Susceptibility Testing guidelines [33]. The following antibiotics (individual antibiotics applied for dedicated strains, according to the guidelines) were used in 2 replications: piperacillin (30 µg), ceftazidime (10 µg), cefiderocol (30 µg), imipenem (10 µg), meropenem (10 µg), tobramycin (10 µg), levofloxacin (5 µg), ciprofloxacin (5 µg), ticarcillin-clavulanic acid (75–10 µg), cefepime (30 µg), amikacin (30 µg), gentamicin (10 µg), moxifloxacin (5 µg), amoxicillin-clavulanic acid (20–10 µg), trimethoprim (5 µg), ampicillin (2 µg), tigecycline (15 µg), linezolid (10 µg), vancomycin (5 µg). Inhibition zone diameters (mm) of each antimicrobial disc were measured and averaged based on repetitions. The isolates were classified as resistant (R), susceptible, increased exposure (I), and susceptible (S). The criteria for selecting species for the antibiotic susceptibility assessment were based on the selection of opportunistic pathogens posing potential threats to public health safety. The criteria for antibiotic selection were based on selecting representativeness for a broad spectrum of antibiotic classes.

## 3. Results

### 3.1. Species Identification

The results demonstrated no growth on the *Legionella* spp. selective medium or *Salmonella–Shigella* Agar. Morphologically distinct colonies observed on other media were subcultured onto fresh media for further species identification. The results demonstrated the presence of both susceptible and resistant bacterial species. The isolated microorganisms belonged to diverse bacterial genera. Identification using mass spectrometry demonstrated the isolation of diverse species representing families: *Staphylococcaceae*, *Lactobacillaceae*, *Micrococcaceae*, *Enterobacteriaceae*, *Enterococcaceae*, *Pseudomonadaceae*, *Alcaligenaceae*, *Flavobacteriaceae*, *Comamonadaceae*, and *Morganellaceae* (Table 2). A variety of bacterial species, including *Staphylococcus sciuri*, *Micrococcus luteus*, *Lelliottia amnigena*, and *Enterococcus casseliflavus*, were present in the wash water samples collected from location A. *Staphylococcus sciuri* bacteria were isolated in both cucumber and plum washing water samples. At the onion processing plant facility (B), differentiated bacteria, including indicators of fecal contamination, were also found. Location C demonstrated, among others, *Pediococcus pentosaceus*, *Enterobacter ludwigii*, and *Micrococcus luteus*, as well as bacteria requiring increased preventive control. Water samples from plant D identified bacteria such as *Lelliottia amnigena*, *Pseudomonas putida*, *Staphylococcus equorum*, *Proteus vulgaris*, *Empedobacter falsenii*, and *Providencia alcalifaciens*. The detected bacteria also included opportunistic pathogens of clinical importance (*Enterococcus faecalis*, *Pseudomonas aeruginosa*, *Klebsiella oxytoca*, *Klebsiella pneumoniae*, *Serratia marcescens*, and *Proteus vulgaris*).

**Figure 3:**
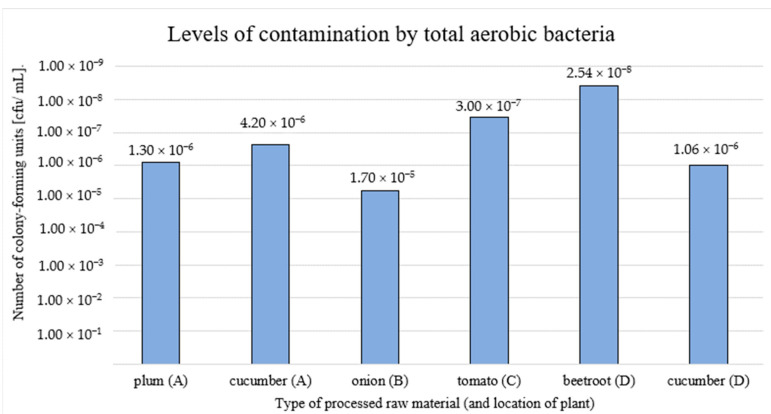
Levels of microbiological contamination of water after fruit and vegetable wash processes (A, B, C, D—locations of fresh-cut processing plants).

The analysis results of bacterial contamination levels in the washing waters varied (Figure 3). The highest level of contamination reached 2.54 × 10^8^ cfu/mL (sample taken after flushing of beetroot; location D). The lowest level of contamination was 1.70 × 10^5^ cfu/mL at location B (sample collected after onion wash).

### 3.2. Antibiotic Susceptibility

Six bacterial strains obtained from the selected samples were tested: *Enterococcus faecalis* (isolation: site B; type of washed raw material: onions), *Pseudomonas aeruginosa* (isolation: site D; type of washed raw material: cucumbers), *Klebsiella oxytoca* (isolation: site C; type of washed raw material: tomatoes), *Klebsiella pneumoniae* (isolation: site B; type of washed raw material: onions), *Serratia marcescens* (isolation: site C; type of washed raw material: tomatoes), and *Proteus vulgaris* (isolation: site D; type of washed raw material: beetroots) (Table 3).

The *P. aeruginosa* isolate was observed to exhibit susceptibility to cefiderecol, meropenem, tobramycin, and amikacin. Intermediate levels (susceptible, but with increased exposure) were also found for piperacillin, ceftadizime, imipenem, levofloxacin, ciprofloxacin, ticarcillin-clavulanic acid, and cefepime.

*E. faecalis* strains demonstrated susceptibility to all the antibiotics tested. Susceptibility with increased exposure was found for imipenem, with an inhibition zone diameter of 30.5 mm. For moxifloxacin, there are no clinical breakpoints, but acquired resistance should be excluded (when acquired resistance is excluded, the isolate should be reported as “devoid of fluoroquinolone resistance mechanisms” but not as susceptible to moxifloxacin).

Testing the antimicrobial susceptibility of *K. oxytoca* rods to 15 different antibiotics also revealed susceptibility to most of the applied agents. The tested bacteria exhibit resistance to cefepime (inhibition zone: 8 mm) and susceptibility with increased exposure to imipenem. The testing results for the *K. pneumoniae* strain indicated susceptibility with increased exposure to imipenem (33.5 mm). Examination of other agents belonging to six antibiotic classes resulted in the absence of phenotypic bacterial resistance. For cefepime, no resistance was found; the zone of inhibition reached 38 mm. The *S. marcescens* strain demonstrated susceptibility with increased exposure to agents from three antibiotic classes: imipenem (32.5 mm), tobramycin (19.5 mm), and amoxicillin-clavulanic acid (19.5 mm). The isolate tested was susceptible to all the remaining antibiotics. The results revealed that the *P. vulgaris* strain was generally also susceptible to all antibiotics tested. Similarly to the previous results, the isolate was classified as imipenem-susceptible with increased exposure.

## 4. Discussion

Research on microbial contamination and cross-contamination of minimally processed fruit and vegetables has focused the attention of researchers for years. There are limited studies that have explored bacterial isolation from wash waters, with additional assessment of phenotypic antibiotic resistance, conducted in several diverse fruit and vegetable processing plants. The current study addresses this gap by conducting a thorough analysis across different facilities, offering a broader perspective on the presence and resistance patterns of bacteria in these environments. The water recycling and implementation of the closed-loop economy principles are recognized as an essential aspect of food processing and a part of a sustainable development strategy. However, there is a consensus highlighting that recycled water must meet high quality standards to ensure the safety of the final product [34].

Regarding the levels of microbial contamination with total bacterial counts, our results revealed relatively low and medium levels of contamination, compared to previous studies, which reported contamination levels of up to 5 to 9 log units [8,34,35]. The results correlate with an earlier assessment of microbial contamination at an apple processing unit, where a total contamination level of 4 log units was obtained [7].

Differences in microbial contamination levels are explained by variable factors related to geographical location, type of raw material processed, technological process, disinfection approach, season, etc. As noted by Zhou et al., secondary bacterial multiplication is linked to the presence of post-harvest residues and damaged vegetables in the water, which may be the main driver of bacterial contamination [36,37].

The identification of bacterial species via mass spectrometry, mostly applied in clinical microbiological investigations, indicates the presence of bacteria of diverse origins. In the current study, the isolated strains were soil bacteria, fecal contamination indicators, as well as plant and human pathogenic bacteria. The findings correlate with an investigation by Liu et al. on species analysis of the production environment in a fresh-cut processing plant. The study found the presence of high bacterial biodiversity originating from various sources [38].

*Staphylococcus* spp., represented in our study by *Staphylococcus equorum* and *Staphylococcus sciuri*, confirm previously reported detections of representatives of this bacterial family. An investigation by Sun et al. on the microbial diversity of fresh-cut lettuce during processing and storage demonstrated the occurrence of pathogenic *S. aureus* [39]. This supports the idea that inadequate washing of fresh-cut vegetables and fruits can lead to contamination, which continues during transportation and distribution. It is important to note that *S. equorum* (coagulase-negative staphylococci) are frequently found in both processing units and various foods, including ready-to-eat products [40,41]. *S. sciuri* bacteria, on the other hand, may present a specific threat due to its potential opportunistic pathogenicity [42]. Given that it is principally an animal-associated bacterial species (inhabiting, e.g., the skin of free-living rodents), it is possible to indicate that the raw material (in the case of our study, cucumbers) may have been subjected to potential animal exposure [43].

The current study also indicates frequent isolation of members of the *Enterobacteriaceae* family, including typical opportunistic pathogens. Research conducted by Pintor-Cora et al. suggests that contamination with *Enterobacteriaceae* representatives occurs as early as during cultivation. *Enterobacteriaceae* rods, exceeding the detection limit, were found in 82.9% of vegetable samples and 36.8% of environmental samples (study based on 117 vegetable samples and 57 farm locations) [44]. The detected isolates of *K. oxytoca* and *K. pneumoniae* may originate from soil, natural fertilizer, and water, owing to the widespread presence of *Klebsiella* spp. in the natural environment [45].

The detection of *K. pneumoniae* in food products is alarming since it is classified among the ESKAPE bacteria group. Opportunistic infections caused by the above pathogens can include urinary tract infections, pneumonia, liver abscesses, bacteremia, soft tissue infections, endophthalmitis, and meningitis [46]. Liu et al. emphasize the biofilm-forming potential of *K. pneumoniae* rods in the processing environment of fresh fruits and vegetables. This could pose a threat due to the bacterium’s ability to persist and propagate for extended periods despite disinfection measures [38]. Other strains of the *Enterobacteriaceae* family isolated in our study, such as *P. vulgaris* and *S. marcescens*, could also pose threats to public health and safety. Hence, their presence in the environment of fresh-cut vegetable and fruit processing facilities is undesirable and therefore should be constantly monitored and kept to a minimum [47,48].

The presence of *Enterococcus* spp. in the wash water indicates most often fecal contamination, as these bacteria are commonly found in such environments. It is consistent with our earlier studies, which also demonstrated the presence of *Enterococcus* spp. in wash water, indicating the widespread fecal contamination of fresh vegetables and fruits [7]. The distribution and ecology of the *E. casseliflavus* isolate are less understood compared to *E. faecalis*; however, the pathogen also colonizes the human intestinal tract due to the regularity of its isolation in nosocomial infections with vancomycin-resistant strains [49]. The problematic nature of *Enterococcus* spp. is due to both natural and easily acquired and maintained resistance to a broad spectrum of antibiotics [50]. Research by Xie et al. also indicates the ease of transfer of these pathogens due to workers’ loss of hygiene, particularly during the step of packaging of fresh fruit and vegetables [51].

Our study also revealed the presence of representatives of *Pseudomonas* spp. in the tested water samples. While *P. putida*, a rhizosphere-borne classical root colonizer, does not pose a major threat to vegetable and fruit sanitization processes, the detection of *P. aeruginosa* may provide additional production challenges. Due to their ubiquity, *P. aeruginosa* bacteria are also already isolated in final fresh-cut products, tolerating well the washing and pre-processing procedures of the raw material [52,53]. It is noteworthy that *P. aeruginosa* intercalates into plant tissues and can colonize them for long periods without visible disease symptoms (or manifesting soft-rot symptoms) [54]. Due to its genome plasticity, broad adaptability, high biofilm production capacity, as well as advanced secretion systems, *P. aeruginosa* is regarded as a significant pathogen. The bacterium is particularly concerning since it causes several infections that include nosocomial pneumonia, surgical wound infections, urinary tract infections, and bacteremia [55,56].

The second part of this study, related to the assessment of the susceptibility of selected isolates to various antibiotics, revealed the resistance of *K. oxytoca* to the application of cefepime. An increase in antibiotic resistance level in clinical strains may have a substantial impact on the spread of resistance in environmental strains. Clinical studies on resistance trends in *K. pneumoniae* causing urinary tract infections, conducted on 1543 *K. pneumoniae* isolates from 2011 to 2019, have shown a notable rise in cefepime resistance levels. Specifically, the level of resistance to cefepime increased from 18.2% to 30.5% by 2017 [57]. As noted by Okaiyeto et al., the contamination of food chains by antibiotic-resistant bacteria is a growing global problem that requires enhanced action and efforts to implement an integrated approach. This strategy should include not only preventive measures but also novel monitoring systems, along with the provision of antibacterial agents [58]. Noteworthy is that cefepime can be degraded by some extended-spectrum β-lactamases (ESBLs) and carbapenemases (although it shows moderate resistance to hydrolysis by OXA-48) [59,60]. In this context, it is also crucial to control the transfer of antibiotics to the environment. As noted by Wang et al., exposure to low concentrations of cefepime can lead to significant antimicrobial resistance levels in environmental bacteria [61]. Overall, the low detection rate of phenotypic resistance is encouraging, particularly considering the numerous reports of AMR strains found on fresh fruits and vegetables, where washing waters can serve as a source of contamination. It is important to highlight that geographical location should not be overlooked in this context. Research by Salmanov et al. (Ukraine) demonstrated the isolation of various concerning pathogens, such as methicillin-resistant *S. aureus*, vancomycin-resistant *Enterococcus* spp., and third-generation cephalosporin-resistant *K. pneumoniae* and *E. coli* [62]. Saksena et al. (India) also highlight the frequent isolation of AMR coliforms from fresh vegetables and fruits, exhibiting ESBLs and carbapenem resistance [63]. As noted by Tiedje et al., the food production system may be an underestimated reservoir for antibiotic-resistant bacteria and antibiotic-resistance genes [64]. An example is the research conducted on the analysis of contamination of vegetables by *Pseudomonas* spp. in Jamaica. In antimicrobial susceptibility tests, it was found that isolates were resistant or had reduced susceptibility to ampicillin, chloramphenicol, sulfamethoxazole/trimethoprim, and aztreonam, and up to 35% were resistant to four different antibiotics [65].

A limitation of this study is that the MALDI Biotyper is primarily designed for the identification of clinical strains, which led to the inability to identify four species. Nonetheless, this study is relevant to public health safety aspects, such as monitoring and controlling pathogens and identifying potential threats.

In summary, our research contributes to understanding the microbiological contamination of minimally processed vegetables and fruits and underscores the importance of adhering to high sanitary standards. The isolated bacteria, including potentially pathogenic ones, pose a challenge to health safety. The findings from our study can contribute to further actions aimed at improving hygiene and safety in food production. Future research could explore the impact of various disinfection methods on microbiological contamination in wash waters. An interesting area of investigation is the use of non-invasive biological methods, which, by reducing the reliance on traditional chemical methods, could help mitigate microbiological risks and support sustainable food production [66].

## 5. Conclusions

In conclusion, bacterial species identification studies carried out by mass spectrometry have shown that the wash waters of fresh-cut processing plants can be a constant habitat for a diverse range of bacteria.

Opportunistic pathogens such as *Enterococcus faecalis*, *Pseudomonas aeruginosa*, *Klebsiella oxytoca*, *Klebsiella pneumoniae*, *Serratia marcescens*, and *Proteus vulgaris* were found among the isolated strains. The isolation of cefepime-resistant *K. oxytoca* indicates that the waters of the agri-food industry may be considered a site for the potential development and maintenance of antibiotic resistance. Broader research is needed to investigate the mechanisms of bacterial contamination in washing water further and develop more effective sanitation methods. If contaminated washing water is not properly managed, potential risks to consumers include contamination of food chains and the consequent threat to consumer safety. These findings highlight the importance of monitoring the microbial microflora in minimally processed foods and the need for appropriate sanitary control procedures to minimize the risk of pathogen contamination.

## Figures and Tables

**Table 1:** Summary of vegetables and fruit types processed in the investigated units.

Plant	Type of Processed Fruit/Vegetable	Type of Final Product
A	strawberries, raspberries, cherries, currants, rhubarb, plums, apples, tomatoes, cucumbers, leeks, broccoli, cauliflower	ready-to-eat fruits and vegetables, frozen products
B	onions	peeled onion, onion rings
C	tomatoes, apples	ready-to-eat fruits and vegetables, apple and tomato concentrate
D	beetroots, cucumbers, onions	ready-to-eat fruits and vegetables, pickled cucumbers, vegetable salads

Abbreviations: A, B, C, D—locations of fresh-cut processing plants.

**Table 2:** Results of bacterial species identification.

No.	Location	Raw Material Type	Species
1	A	cucumber	*Staphylococcus sciuri*
2	A	cucumber	*Micrococcus luteus*
3	A	plum	*Staphylococcus sciuri*
4	A	cucumber	*Lelliottia amnigena*
5	A	cucumber	*Enterococcus casseliflavus*
6	A	cucumber	*Comamonas testosteroni*
7	B	onion	*Enterobacter ludwigii*
8	B	onion	*Kerstersia gyiorum*
9	B	onion	*Citrobacter braakii*
10	B	onion	*Pseudomonas aeruginosa*
11	B	onion	*Enterococcus faecalis*
12	B	onion	*Klebsiella pneumoniae*
13	C	tomato	*Pediococcus pentosaceus*
14	C	tomato	*Enterobacter ludwigii*
15	C	tomato	*Micrococcus luteus*
16	C	tomato	*Klebsiella oxytoca*
17	C	tomato	*Pseudomonas protegens*
18	C	tomato	*Serratia marcescens*
19	D	cucumber	*Lelliottia amnigena*
20	D	cucumber	*Pseudomonas putida*
21	D	beetroot	*Proteus vulgaris*
22	D	cucumber	*Staphylococcus equorum*
23	D	onion	*Empedobacter falsenii*
24	D	cucumber	*Pseudomonas aeruginosa*
25	D	cucumber	*Providencia alcalifaciens*

Abbreviations: A, B, C, D—locations of fresh-cut processing plants.

**Table 3:** Assessment of antibiotic susceptibility profiles of the investigated bacteria.

Type of Antibiotic		Antibiotic Susceptibility Profiles of Bacteria					
Class	Antibiotic	*K. oxytoca*	*K. pneumoniae*	*S. marcescens*	*P. vulgaris*	*P. aeruginosa*	*E. faecalis*
Penicillins	piperacillin	S	S	S	S	I	n. a.
Penicillins	amoxicillin-clavulanic acid	S	S	n. a.	S	n. a.	n. a.
Penicillins	ticarcillin-clavulanic acid	S	S	S	S	I	S
Penicillins	ampicillin	n. a.	n. a.	n. a.	n. a.	n. a.	S
Cephalosporins	cefepime	R	S	S	S	I	n. a.
Cephalosporins	cefiderocol	S	S	S	S	S	n. a.
Cephalosporins	ceftazidime	S	S	S	S	I	n. a.
Carbapenems	meropenem	S	S	S	S	S	n. a.
Carbapenems	imipenem	I	I	I	I	I	I
Fluoroquinolones	ciprofloxacin	S	S	S	S	I	S
Fluoroquinolones	moxifloxacin	S	S	n. a.	S	n. a	*
Fluoroquinolones	levofloxacin	S	S	S	S	I	S
Aminoglycosides	gentamicin	S	S	S	S	n. a.	n. a.
Aminoglycosides	amikacin	S	S	S	S	S	n. a.
Aminoglycosides	tobramycin	S	S	I	S	S	n. a.
Chemotherapeutics	trimethoprim	S	S	S	S	n. a.	n. a.
Chemotherapeutics	trimethoprim	n. a.	n. a.	n. a.	n. a.	n. a.	S
Glycopeptides	vancomycin	n. a.	n. a.	n. a.	n. a.	n. a.	S
Tetracyclines	tigecycline	n. a.	n. a.	n. a.	n. a.	n. a.	S
Oxazolidinones	linezolid	n. a.	n. a.	n. a.	n. a.	n. a.	S

Abbreviations: resistant: R; intermediate: I (susceptible, increased exposure); susceptible: S; n. a.—not applicable; *—devoid of fluoroquinolone resistance mechanisms.

## Data Availability

All relevant data supporting the findings of this article are included within the manuscript. Raw data, as well as any additional materials, are available upon request from the corresponding author.
